# Adverse events of pexidartinib for the treatment of TGCT: a real-world disproportionality analysis using FDA Adverse Event Reporting System database

**DOI:** 10.3389/fonc.2025.1594585

**Published:** 2025-08-18

**Authors:** Yu Lin, Xinlei Zheng, Li Lin, Maohua Chen

**Affiliations:** ^1^ Department of Orthopedics, Fujian Medical University Union Hospital, Fuzhou, China; ^2^ Department of Pharmacy, Pingtan Comprehensive Experimental Area Hospital, Fuzhou, China; ^3^ Department of Medical Oncology, Department of Medical Oncology, Shengli Clinical Medical College of Fujian Medical University, Fujian Provincial Hospital, Fuzhou University Affiliated Provincial Hospital, Fuzhou, Fujian, China

**Keywords:** disproportionality analysis, FAERS database, pexidartinib, real-world adverse events, tenosynovial giant cell tumor

## Abstract

**Introduction:**

Pexidartinib, an oral selective colony-stimulating factor 1 receptor (CSF1R) inhibitor, is the only systemic therapy approved by the U.S. Food and Drug Administration (FDA) for tenosynovial giant cell tumor (TGCT). While clinical trials have defined its initial safety profile, their limited sample sizes and short follow-up restrict the detection of rare or delayed adverse events (AEs), underscoring the need for real-world pharmacovigilance.

**Methods:**

Using the FDA Adverse Event Reporting System (FAERS) database, we conducted a disproportionality analysis to characterize the post-approval safety profile of pexidartinib and identify unlabeled AEs, applying reporting odds ratio (ROR), proportional reporting ratio (PRR), Bayesian confidence propagation neural network (BCPNN), and multi-item gamma Poisson shrinker (MGPS) methods.

**Results:**

Among 7,168,342 FAERS reports, 668 implicated pexidartinib as the primary suspect, with AEs reported across 26 organ systems. Sixty-seven preferred terms met the criteria of all four signal detection methods, including 16 not listed in the FDA-approved label.

**Discussion:**

The overall safety profile was largely consistent with clinical trial findings, while newly detected AEs suggest possible rare or delayed toxicities in broader patient populations. These results highlight the importance of continuous post-marketing surveillance and support the need for prospective studies to clarify causal relationships.

## 1 Introduction

Tenosynovial giant cell tumor (TGCT), historically termed pigmented villonodular synovitis (PVNS) or giant cell tumor of the tendon sheath, is a rare mesenchymal neoplasm originating from synovial membranes of joints and the tendon sheaths (1). For patients with symptomatic TGCT refractory to local therapies or causing significant functional impairment, systemic therapy should be considered if surgery is unlikely to achieve functional outcome improvement. The molecular pathogenesis of TGCT involves genomic alterations at the colony-stimulating factor 1 (CSF1) locus (1p13), resulting in aberrant CSF1 overexpression in a subset of tumor cells (2). This molecular pathology supports CSF1/CSF1 receptor (CSF1R) axis inhibition as a central therapeutic strategy.

Pexidartinib, an oral selective CSF1R inhibitor, represents the only approved systemic treatment for TGCT (3). Its Food and Drug Administration (FDA) approval in August 2019 introduced it as the first systemic treatment for adult patients with symptomatic TGCT associated with severe morbidity or functional limitations and not amenable to improvement with surgery (4, 5). Pre-marketing clinical trials have since validated pexidartinib’s efficacy and safety, reinforcing its pivotal role in TGCT management (6, 7).

Comprehensive monitoring of pexidartinib’s real-world safety profile remains imperative given its expanding clinical utilization following FDA approval. Drug safety assessments advocate intervention strategies, including dose control, to mitigate toxicity (8). According to FDA’s prescribing information, pexidartinib’s common adverse events (AEs) included increased lactate dehydrogenase, increased aspartate aminotransferase, hair color changes, fatigue, increased alanine aminotransferase, decreased neutrophils, increased cholesterol, increased alkaline phosphatase, decreased lymphocytes, periorbital edema, decreased hemoglobin, rash, dysgeusia, and decreased phosphate. However, patients may experience AEs during off-label use due to comorbidities, concomitant medications, or genetic predispositions. Given the limited sample sizes and short observation periods in clinical trials, extensive post-marketing safety research in real-world settings is essential. Nevertheless, comprehensive real-world pharmacovigilance studies specifically addressing its AEs remain notably lacking.

The FDA Adverse Event Reporting System (FAERS) serves as a national post-marketing surveillance tool and one of the world’s largest pharmacovigilance databases, designed to systematically capture spontaneous safety reports for therapeutic agents (9, 10). Functioning as a critical spontaneous reporting system, this continuously updated, quarterly database (11) captures diverse safety signals, including clinician-reported AEs, medication error documentation, and product quality complaints. For the past few years, many drug safety profile studies based on the FAERS database have been published, affirming the reliability of this resource (12, 13). Using disproportionality analysis based on the FAERS database, we identified post-marketing safety signals associated with pexidartinib. This study detected AEs not previously documented in FDA-approved prescribing information. Importantly, these findings provide critical insights for optimizing therapeutic monitoring and enhancing pharmacovigilance strategies in clinical practice.

## 2 Patients and methods

### 2.1 Data sources and procedures

Available data related to pexidartinib were extracted and analyzed from the FAERS database (https://fis.fda.gov/extensions/FPD-QDE-FAERS/FPD-QDE-FAERS.html). There was no need for specific ethical approval and informed consent, as no direct human intervention or human sample collection was required.

The FAERS dataset comprises seven sections containing demographic and management information, drug information, adverse drug reaction information, patient outcomes, reporting sources, start and end dates of treatment with reported drugs, indications, and deleted cases. Since pexidartinib was approved in August 2019, we included data from the third quarter of 2019 through the second quarter of 2023. Given the potential for duplicate entries in the FAERS database, we performed a rigorous deduplication process. Specifically, we manually reviewed reports to remove entries with lower PRIMARYIDs when the CASEIDs are the same. Moreover, we further eliminated records listed in the deleted case file. We then identified pexidartinib-associated cases in both the “drugname” and “prod_ai” columns using “pexidartinib” and “SUNOSI” in the “DRUG” files.

Pexidartinib-related cases were identified in both the “drugname” and “prod_ai” fields of the DRUG file using the terms “pexidartinib” and “SUNOSI”. Adverse event dates (EVENT_DT) were obtained from the DEMO file, and therapy start dates (START_DT) were obtained from the THER file. Time-to-onset (TTO) was calculated as the difference between EVENT_DT and START_DT. Records were included only if both dates were valid and correctly formatted (YYYYMMDD), and reports with incomplete dates or implausible sequences (i.e., EVENT_DT earlier than START_DT) were excluded. TTO analysis was conducted based on medians, quartiles, and the Weibull shape parameter (WSP) test. The WSP analysis was conducted using the Minitab statistical software (v20.0; Minitab LLC, State College, PA, USA).

All reported AEs were coded using the Medical Dictionary for Regulatory Activities version 26.0 (MedDRA 26.0). The MedDRA terminology is structured hierarchically into five levels: system organ class (SOC), high-level group term (HLGT), high-level term (HLT), preferred term (PT), and lowest-level term (LLT). This study focused on identifying all pexidartinib-related AEs recorded in the adverse reaction files of FAERS. Events were systematically classified and analyzed at both the SOC and PT levels to assess their distribution and severity.

In FAERS, the drug role is designated by the reporter using one of three codes: 1 means primary suspect, 2 means concomitant, and 3 means interacting. To improve analytical specificity, we included only records in which pexidartinib was designated as the primary suspect (code 1). This strategy was adopted to enhance the accuracy and reliability of the safety signal detection.

### 2.2 Statistical analysis

Disproportionality analysis is a crucial technique in pharmacovigilance studies, serving a vital role in identifying potential signals indicating AEs associated with a drug (14). This methodology involves a comparative assessment of the frequency of AEs linked to a specific drug relative to the occurrence of AEs related to all other medications. Fundamentally, it relies on the concept that a signal emerges during data extraction when the incidence rate of a particular AE for a given drug significantly exceeds the background occurrence rate observed across the entire database. This deviation from the norm must exceed a predetermined threshold or set of criteria to be considered statistically significant.

In our analysis, we employed both frequentist and Bayesian approaches within the framework of disproportionality analysis. This dual approach enabled us to explore the association between a drug and a specific AE. We used the reporting odds ratio (ROR), proportional reporting ratio (PRR), Bayesian confidence propagation neural network (BCPNN), and multi-item gamma Poisson shrinker (MGPS) algorithms to quantify the signals of pexidartinib-related AEs (15).

In this study, a signal was considered valid only when the criteria of all four algorithms were simultaneously met. For further details regarding the mathematical equations and specific threshold values for each algorithm, the readers are referred to **Supplementary Table S1**. All disproportionality measures were derived from a standard 2 × 2 contingency table, as shown in **Supplementary Table S2**.

## 3 Results

### 3.1 Characteristics of AE reports

**Figure 1:**
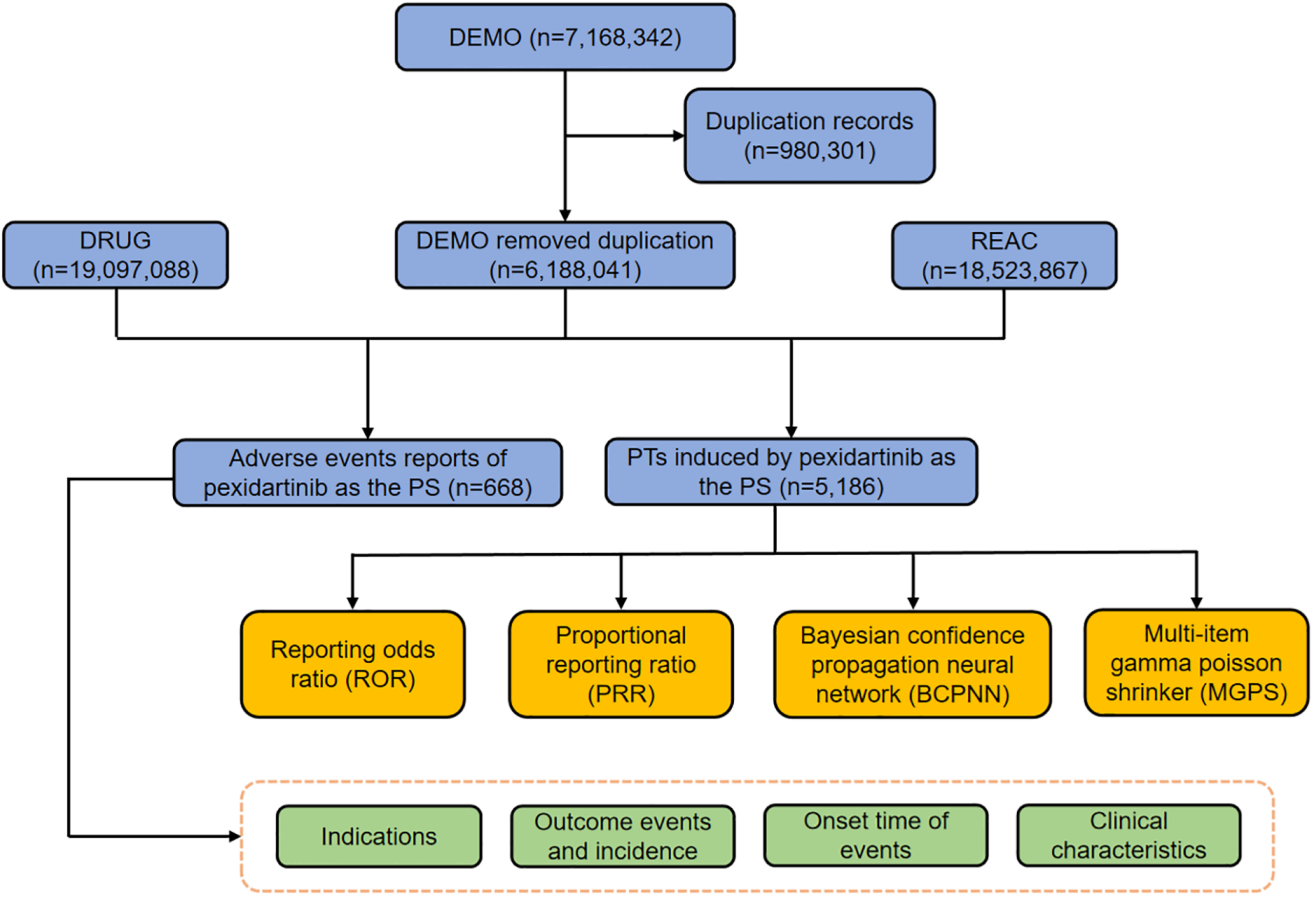
The flow diagram of selecting pexidartinib-related AEs from FAERS database. DEMO, demographic file; DRUG, drug file; REAC, reaction file; PS, primary suspect; AEs, adverse events; FAERS, Food and Drug Administration Adverse Event Reporting System.

Throughout the course of the study, a total of 7,168,342 AE reports were initially scrutinized. Following meticulous deduplication of duplicate entries, a refined dataset of 668 reports directly associated with pexidartinib remained, as depicted in **Figure 1**.

To characterize patients who experienced AEs related to pexidartinib, their baseline demographics and clinical features are summarized in **Table 1**. Of the patients who experienced AEs linked to pexidartinib collected from FAERS, the majority was female (n = 340, 60.18%), adult (n = 161, 84.29%), and patients with TGCT or PVNS (n = 468, 82.69%). Our analysis of the top five co-administered drugs in pexidartinib-related AE cases identified amlodipine, vitamin D3, famotidine, ondansetron (Zofran), and oxycodone as the most frequently reported agents. Regarding serious clinical outcomes, hospitalization was the most frequently reported (n = 64, 41.56%), followed by death (n = 13, 8.44%). The median TTO of AEs was 20 days (interquartile range: 6.5–211 days). With the exception of the first and second quarters of 2023 and the third and fourth quarters of 2019, the highest number of AE reports was recorded in 2022 (n = 190).

**Table 1:** Clinical characteristics of reports with pexidartinib from the FAERS database.

Characteristics	Pexidartinib-induced AE reports (n = 668)		
Number of events	Available number, n	Case number, n	Case proportion, %
Gender, n (%)	565	–	84.58%
Female	–	340	60.18%
Male	–	225	39.82%
Age (years), n (%)	191	–	28.59%
<18	–	7	3.66%
18≤ and ≤65	–	161	84.29%
>65	–	23	12.04%
Median [Interquartile Range(IQR)]	–	45.5 (33.5–59.5)	–
Weight (kg), n (%)	65	–	9.73%
<80	–	31	47.69%
80≤ and ≤100	–	23	35.38%
>100	–	11	16.92%
Median (IQR)	–	80.27 (65.76–93.42)	–
Reported countries, n (%)	668	–	100.00%
United States (US)		667	99.85%
Italy (IT)		1	0.15%
Indications, n (%)	566	–	84.73%
Giant cell tumor of tendon sheath	–	257	45.41%
Synovitis		211	37.28%
Others	–	98	17.31%
Combination drugs, n (%)	164	–	24.55%
Amlodipine	–	15	9.15%
Vitamin D3	–	14	8.54%
Famotidine	–	11	6.71%
Zofran	–	10	6.10%
Oxycodone	–	10	6.10%
Outcomes, n (%)	668	–	100.00%
Non-serious outcome	–	514	76.95%
Serious outcome	–	154	23.05%
Death	–	13	8.44%
Life-threatening	–	3	1.95%
Hospitalization	–	64	41.56%
Disability	–	2	1.30%
Other serious outcomes	–	104	67.53%
Time to onset (days)	23		3.44%
Median (IQR)		20 (6.5–211)	
Reporters, n (%)	668	–	100.00%
Health professional	–	367	54.94%
Consumer	–	301	45.06%
Reporting year, n (%)	668	–	100.00%
2023 Q1–Q2	–	190	28.44%
2022	–	190	28.44%
2021	–	133	19.91%
2020	–	145	21.71%
2019 Q3–Q4	–	10	1.50%

AE, adverse event; FAERS, Food and Drug Administration Adverse Event Reporting System.

### 3.2 Disproportionality analysis

The signal reports for pexidartinib at the SOC level are presented in **Table 2**. Adverse event occurrences linked to pexidartinib encompass a wide spectrum of 26 distinct organ systems. Among these, the most frequently reported SOCs were general disorders and administration site conditions (SOC: 10018065, n = 453), skin and subcutaneous tissue disorders (SOC: 10040785, n = 373), and injury, poisoning, and procedural complications (SOC: 10022117, n = 324). Notably, “skin and subcutaneous tissue disorders” exhibited positive signal detection across all four algorithms. In contrast, “general disorders and administration site conditions” and “injury, poisoning, and procedural complications” generated positive signals only with the PRR method, but not with the ROR, BCPNN, and MGPS methods.

**Table 2:** Signal strength of reports of pexidartinib at the SOC level in FAERS database.

System organ class (SOC)	Pexidartinib cases reporting SOC	ROR (95% two-sided CI)	PRR (χ^2^)	IC (IC025)	EBGM (EBGM05)
General disorders and administration site conditions	453	0.85 (0.77–0.94)	0.87 (10.52)	−0.20 (−0.22)	0.87 (0.79)
Skin and subcutaneous tissue disorders	373	2.57 (2.31–2.87)	2.38 (313.78)	1.25 (1.12)	2.38 (2.13)
Injury, poisoning, and procedural complications	324	0.87 (0.77–0.97)	0.88 (6.06)	−0.18 (−0.21)	0.88 (0.78)
Investigations	304	1.95 (1.73–2.20)	1.86 (126.87)	0.89 (0.79)	1.86 (1.65)
Gastrointestinal disorders	274	1.39 (1.23–1.57)	1.35 (26.98)	0.44 (0.38)	1.35 (1.19)
Nervous system disorders	243	1.16 (1.02–1.32)	1.15 (4.98)	0.20 (0.17)	1.15 (1.01)
Musculoskeletal and connective tissue disorders	158	1.22 (1.04–1.43)	1.21 (6.00)	0.27 (0.23)	1.21 (1.03)
Eye disorders	141	2.71 (2.29–3.21)	2.63 (144.87)	1.39 (1.18)	2.63 (2.22)
Infections and infestations	121	0.70 (0.58–0.84)	0.71 (15.16)	−0.49 (−0.59)	0.71 (0.59)
Psychiatric disorders	91	0.64 (0.52–0.78)	0.65 (18.47)	−0.63 (−0.78)	0.65 (0.52)
Metabolism and nutrition disorders	86	1.29 (1.04–1.60)	1.28 (5.40)	0.36 (0.29)	1.28 (1.03)
Surgical and medical procedures	80	1.51 (1.21–1.88)	1.49 (13.20)	0.58 (0.46)	1.49 (1.19)
Respiratory, thoracic, and mediastinal disorders	57	0.44 (0.34–0.58)	0.46 (38.81)	−1.14 (−1.48)	0.46 (0.35)
Vascular disorders	46	0.65 (0.48–0.86)	0.65 (8.82)	−0.62 (−0.83)	0.65 (0.49)
Hepatobiliary disorders	36	1.28 (0.92–1.78)	1.28 (2.17)	0.35 (0.25)	1.28 (0.92)
Renal and urinary disorders	34	0.56 (0.40–0.78)	0.56 (11.68)	−0.83 (−1.16)	0.56 (0.40)
Immune system disorders	34	0.74 (0.52–1.03)	0.74 (3.20)	−0.44 (−0.61)	0.74 (0.53)
Blood and lymphatic system disorders	29	0.51 (0.35–0.73)	0.51 (13.81)	−0.97 (−1.40)	0.51 (0.35)
Neoplasms benign, malignant, and unspecified (incl cysts and polyps)	27	0.17 (0.12–0.25)	0.18 (105.87)	−2.47 (−3.61)	0.18 (0.12)
Reproductive system and breast disorders	27	1.27 (0.87–1.86)	1.27 (1.57)	0.35 (0.24)	1.27 (0.87)
Social circumstances	14	0.76 (0.45–1.28)	0.76 (1.06)	−0.39 (−0.67)	0.76 (0.45)
Ear and labyrinth disorders	11	0.71 (0.40–1.29)	0.72 (1.25)	−0.48 (−0.87)	0.72 (0.40)
Cardiac disorders	10	0.15 (0.08–0.28)	0.15 (48.11)	−2.71 (−5.05)	0.15 (0.08)
Product issues	9	0.14 (0.07–0.26)	0.14 (48.51)	−2.83 (−5.45)	0.14 (0.07)
Endocrine disorders	7	0.73 (0.35–1.52)	0.73 (0.72)	−0.46 (−0.97)	0.73 (0.35)
Pregnancy, puerperium, and perinatal conditions	2	0.17 (0.04–0.68)	0.17 (8.15)	−2.56 (−10.23)	0.17 (0.04)

SOC, system organ class; PT, preferred term; ROR, reporting odds ratio; CI, confidence interval; PRR, proportional reporting ratio; χ^2^, chi-information component; IC, information component; IC025, the lower limit of 95% CI of the IC; EBGM, empirical Bayesian geometric mean; EBGM05, the lower limit of 95% CI of EBGM.

Additionally, disproportionality analysis was performed at the PT level, as presented in **Table 3**. A comparison was made between the detected PTs and the adverse reactions listed in the official prescribing information, with an asterisk (*) used to indicate events not mentioned in the label. The unlabeled AEs not previously documented in FDA-approved information included photosensitivity reaction, dysmenorrhea, oligomenorrhea, increased gamma-glutamyltransferase, decreased blood iron, increased blood calcium, prolonged prothrombin time, increased red cell distribution width, hepatitis A, seasonal allergy, vanishing bile duct syndrome, cholecystitis, hunger, soft feces, eye color change, and blepharospasm.

**Table 3:** Top significant signals on the PT level.

SOC	Preferred terms (PTs)	Pexidartinib cases reporting PT	ROR (95% two-sided CI)	PRR (χ^2^)	IC (IC025)	EBGM (EBGM05)
Surgical and medical procedures	Therapy cessation	20	3.66 (2.36–5.68)	3.65 (38.45)	1.87 (1.20)	3.65 (2.35)
Surgical and medical procedures	Therapy change	9	10.77 (5.59–20.73)	10.75 (79.38)	3.42 (1.78)	10.72 (5.57)
Surgical and medical procedures	Tumor excision	3	46.40 (14.85–144.98)	46.37 (131.48)	5.52 (1.77)	45.79 (14.66)
Skin and subcutaneous tissue disorders	Hair color changes	238	205.28 (179.64–234.59)	195.91 (43,761.88)	7.54 (6.60)	185.77 (162.57)
Skin and subcutaneous tissue disorders	Pruritus	123	4.09 (3.42–4.89)	4.02 (280.14)	2.01 (1.68)	4.01 (3.36)
Skin and subcutaneous tissue disorders	Skin discoloration	38	10.08 (7.32–13.87)	10.01 (307.55)	3.32 (2.41)	9.99 (7.25)
Skin and subcutaneous tissue disorders	Photosensitivity reaction*	14	10.67 (6.31–18.05)	10.65 (122.02)	3.41 (2.02)	10.62 (6.28)
Skin and subcutaneous tissue disorders	Sensitive skin	8	6.37 (3.18–12.74)	6.36 (36.07)	2.67 (1.33)	6.35 (3.17)
Skin and subcutaneous tissue disorders	Skin hypopigmentation	6	27.68 (12.39–61.82)	27.64 (152.91)	4.78 (2.14)	27.44 (12.28)
Skin and subcutaneous tissue disorders	Pigmentation disorder	5	9.43 (3.92–22.70)	9.43 (37.57)	3.23 (1.34)	9.40 (3.91)
Skin and subcutaneous tissue disorders	Rosacea	3	7.59 (2.44–23.57)	7.59 (17.12)	2.92 (0.94)	7.57 (2.44)
Reproductive system and breast disorders	Dysmenorrhea*	5	5.46 (2.27–13.14)	5.46 (18.19)	2.45 (1.02)	5.45 (2.27)
Reproductive system and breast disorders	Oligomenorrhea*	3	32.88 (10.55–102.51)	32.86 (91.83)	5.03 (1.61)	32.57 (10.45)
Nervous system disorders	Taste disorder	33	10.59 (7.51–14.91)	10.52 (283.79)	3.39 (2.41)	10.50 (7.45)
Nervous system disorders	Dysgeusia	27	5.65 (3.87–8.25)	5.62 (102.61)	2.49 (1.71)	5.62 (3.85)
Nervous system disorders	Ageusia	15	7.55 (4.54–12.54)	7.53 (84.78)	2.91 (1.75)	7.52 (4.52)
Nervous system disorders	Brain fog	7	21.00 (9.98–44.16)	20.97 (132.35)	4.38 (2.08)	20.85 (9.91)
Neoplasms benign, malignant, and unspecified (incl cysts and polyps)	Tumor pain	5	40.43 (16.74–97.66)	40.39 (189.95)	5.32 (2.20)	39.95 (16.54)
Metabolism and nutrition disorders	Decreased appetite	57	3.03 (2.33–3.93)	3.00 (76.41)	1.59 (1.22)	3.00 (2.31)
Investigations	Increased aspartate aminotransferase	98	30.23 (24.73–36.94)	29.67 (2,694.65)	4.88 (3.99)	29.44 (24.08)
Investigations	Increased alanine aminotransferase	89	22.23 (18.01–27.43)	21.86 (1,762.62)	4.44 (3.60)	21.74 (17.62)
Investigations	Increased blood alkaline phosphatase	51	40.24 (30.50–53.11)	39.86 (1,911.18)	5.30 (4.02)	39.43 (29.88)
Investigations	Increased gamma-glutamyltransferase*	49	37.72 (28.43–50.04)	37.37 (1,716.95)	5.21 (3.93)	36.99 (27.88)
Investigations	Increased hepatic enzyme	46	7.86 (5.88–10.51)	7.80 (272.29)	2.96 (2.21)	7.78 (5.82)
Investigations	Increased liver function test	34	14.98 (10.69–21.01)	14.89 (438.95)	3.89 (2.78)	14.83 (10.58)
Investigations	Increased blood bilirubin	30	18.57 (12.96–26.62)	18.47 (493.41)	4.20 (2.93)	18.38 (12.83)
Investigations	Abnormal laboratory test	18	7.83 (4.93–12.44)	7.81 (106.61)	2.96 (1.86)	7.79 (4.90)
Investigations	Abnormal liver function test	15	11.99 (7.22–19.92)	11.96 (150.17)	3.58 (2.15)	11.92 (7.18)
Investigations	Increased conjugated bilirubin	12	104.50 (58.82–185.64)	104.26 (1,192.41)	6.66 (3.75)	101.33 (57.04)
Investigations	Abnormal hepatic enzymes	8	19.11 (9.53–38.30)	19.08 (136.36)	4.25 (2.12)	18.99 (9.47)
Investigations	Decreased blood iron*	8	6.92 (3.46–13.86)	6.91 (40.40)	2.79 (1.39)	6.90 (3.45)
Investigations	Increased blood lactate dehydrogenase	6	6.47 (2.90–14.41)	6.46 (27.64)	2.69 (1.21)	6.45 (2.89)
Investigations	Increased enzyme level	4	46.87 (17.47–125.72)	46.83 (177.09)	5.53 (2.06)	46.24 (17.24)
Investigations	Increased blood calcium*	4	7.64 (2.86–20.37)	7.63 (23.00)	2.93 (1.10)	7.62 (2.85)
Investigations	Prolonged prothrombin time*	3	13.45 (4.33–41.80)	13.44 (34.42)	3.74 (1.20)	13.39 (4.31)
Investigations	Increased red cell distribution width*	3	6.24 (2.01–19.37)	6.24 (13.17)	2.64 (0.85)	6.23 (2.01)
Injury, poisoning, and procedural complications	Product dose omission issue	188	3.79 (3.28–4.39)	3.69 (372.02)	1.88 (1.63)	3.69 (3.19)
Injury, poisoning, and procedural complications	Product dose omission in error	44	16.14 (11.99–21.73)	16.01 (616.72)	3.99 (2.97)	15.94 (11.84)
Injury, poisoning, and procedural complications	Sunburn	11	22.79 (12.59–41.26)	22.74 (227.24)	4.50 (2.49)	22.61 (12.49)
Injury, poisoning, and procedural complications	Wrong dose	4	11.16 (4.18–29.79)	11.15 (36.85)	3.47 (1.30)	11.12 (4.16)
Infections and infestations	Hepatitis A*	5	83.90 (34.55–203.74)	83.82 (399.79)	6.36 (2.62)	81.92 (33.74)
Immune system disorders	Seasonal allergy*	10	6.97 (3.74–12.97)	6.96 (50.92)	2.80 (1.50)	6.94 (3.73)
Hepatobiliary disorders	Vanishing bile duct syndrome*	5	67.44 (27.83–163.43)	67.37 (320.89)	6.05 (2.50)	66.14 (27.29)
Hepatobiliary disorders	Hypertransaminasemia	5	7.57 (3.15–18.21)	7.56 (28.41)	2.92 (1.21)	7.55 (3.14)
Hepatobiliary disorders	Cholecystitis*	4	5.89 (2.21–15.72)	5.89 (16.21)	2.56 (0.96)	5.88 (2.20)
General disorders and administration site conditions	Fatigue	255	4.00 (3.52–4.53)	3.85 (544.21)	1.94 (1.71)	3.85 (3.39)
General disorders and administration site conditions	Feeling abnormal	55	3.01 (2.31–3.92)	2.99 (72.91)	1.58 (1.21)	2.99 (2.29)
General disorders and administration site conditions	No adverse event	41	2.79 (2.05–3.79)	2.78 (46.67)	1.47 (1.08)	2.77 (2.04)
General disorders and administration site conditions	Swelling face	38	8.35 (6.06–11.49)	8.29 (243.41)	3.05 (2.22)	8.28 (6.01)
General disorders and administration site conditions	Disease progression	30	3.01 (2.10–4.31)	3.00 (40.07)	1.58 (1.11)	3.00 (2.09)
General disorders and administration site conditions	Treatment non-compliance	25	6.46 (4.36–9.57)	6.43 (114.61)	2.68 (1.81)	6.42 (4.34)
General disorders and administration site conditions	Thirst	11	7.26 (4.02–13.13)	7.25 (59.15)	2.86 (1.58)	7.24 (4.00)
General disorders and administration site conditions	Facial edema	6	5.59 (2.51–12.45)	5.58 (22.54)	2.48 (1.11)	5.58 (2.50)
General disorders and administration site conditions	Hunger*	5	6.70 (2.78–16.11)	6.69 (24.16)	2.74 (1.14)	6.68 (2.78)
Gastrointestinal disorders	Nausea	145	2.51 (2.13–2.96)	2.47 (128.23)	1.30 (1.11)	2.47 (2.09)
Gastrointestinal disorders	Abdominal discomfort	45	2.96 (2.21–3.97)	2.94 (57.92)	1.56 (1.16)	2.94 (2.19)
Gastrointestinal disorders	Dyspepsia	25	3.56 (2.40–5.27)	3.55 (45.73)	1.83 (1.23)	3.54 (2.39)
Gastrointestinal disorders	Soft feces*	9	11.73 (6.10–22.59)	11.72 (87.94)	3.55 (1.84)	11.68 (6.07)
Gastrointestinal disorders	Abdominal tenderness	3	9.24 (2.97–28.70)	9.23 (21.97)	3.20 (1.03)	9.21 (2.97)
Eye disorders	Periorbital swelling	50	66.78 (50.42–88.46)	66.15 (3,150.39)	6.02 (4.55)	64.97 (49.05)
Eye disorders	Eye swelling	37	13.98 (10.11–19.33)	13.89 (440.99)	3.79 (2.74)	13.84 (10.01)
Eye disorders	Swelling of eyelid	8	9.43 (4.71–18.89)	9.42 (60.07)	3.23 (1.61)	9.40 (4.69)
Eye disorders	Eye edema	8	48.25 (24.00–96.98)	48.17 (364.64)	5.57 (2.77)	47.54 (23.65)
Eye disorders	Periorbital edema	8	23.20 (11.57–46.53)	23.17 (168.61)	4.53 (2.26)	23.03 (11.48)
Eye disorders	Eyelash discoloration	6	766.06 (317.06–1,850.88)	765.17 (3,770.98)	9.30 (3.85)	630.32 (260.88)
Eye disorders	Eye color change*	3	33.50 (10.74–104.44)	33.48 (93.64)	5.05 (1.62)	33.17 (10.64)
Eye disorders	Blepharospasm*	3	7.64 (2.46–23.72)	7.64 (17.26)	2.93 (0.94)	7.62 (2.45)

SOC, system organ class; PT, preferred term; ROR, reporting odds ratio; CI, confidence interval; PRR, proportional reporting ratio; χ^2^, chi-information component; IC, information component; IC025, the lower limit of 95% CI of the IC; EBGM, empirical Bayesian geometric mean; EBGM05, the lower limit of 95% CI of EBGM.

*Not mentioned in the label.

## 4 Discussion

Our pharmacovigilance study provides the first systematic characterization of pexidartinib’s post-marketing safety profile using real-world evidence from the FAERS database. Analysis of AEs’ temporal trends revealed a progressive increase in reports over time, likely attributable to expanded clinical use and enhanced pharmacovigilance awareness following FDA approval. We identified a higher frequency of AE reports among adult women in FAERS, which may reflect the known higher prevalence of TGCT in women. TGCT is more common in women than men, and the mean age at diagnosis is 35–50 years (1, 16). However, due to the absence of an accurate number of patients using pexidartinib, prospective population-based studies with standardized AE ascertainment remain necessary to establish robust risk stratification models.

The results of our study underscore a clustering of common SOCs around “general disorders and administration site conditions”, “injury, poisoning, and procedural complications”, “skin and subcutaneous tissue disorders”, and investigations, as well as gastrointestinal disorders. Additionally, frequently reported PTs associated with pexidartinib include fatigue, hair color changes, product dose omission issues, nausea, pruritus, increased aspartate aminotransferase, and increased alanine aminotransferase. Notably, these AEs are in accordance with information provided in the FDA’s drug label and previous research on pexidartinib. For example, a randomized phase 3 clinical trial of pexidartinib versus placebo for advanced TGCT identified that hair color changes (67%), fatigue (54%), increased aspartate aminotransferase (39%), nausea (38%), increased alanine aminotransferase (28%), and dysgeusia (25%) were the most frequent pexidartinib-associated AEs (6). A study of long-term outcomes of pexidartinib in TGCT revealed that “hair color changes” was the most frequent AE (17). A. Vaynrub et al. concluded that the most common AEs associated with pexidartinib are mild hypopigmentation of the hair and transient aminotransferase elevation (18).

Beyond confirming established safety signals, our pharmacovigilance analysis identified 16 novel adverse drug reactions not currently documented in pexidartinib’s FDA labeling. These include photosensitivity reaction, dysmenorrhea, oligomenorrhea, increased gamma-glutamyltransferase, decreased blood iron, increased blood calcium, prolonged prothrombin time, increased red cell distribution width, hepatitis A, seasonal allergy, vanishing bile duct syndrome, cholecystitis, hunger, soft feces, eye color change, and blepharospasm. Sorbarikor Piawah et al. (19) reported a case of severe drug-induced liver injury requiring liver transplantation due to vanishing bile duct syndrome after exposure to pexidartinib. Beyond vanishing bile duct syndrome previously reported in the literature, most AEs associated with pexidartinib identified in our study represent novel safety findings. These newly detected AEs warrant further validation to ensure the safe clinical use of pexidartinib. Importantly, the mechanisms underlying most unexpected AEs remain unstudied, necessitating dedicated mechanistic investigations.

Occurrences such as therapy cessation, therapy change, tumor excision, product dose omission issue, product dose omission in error, wrong dose, no adverse event, and treatment non-compliance were not classified as pexidartinib-induced AEs. Pexidartinib was often used as therapy for TGCT preoperation and postoperation (20, 21), and a few patients have reported drug dosage reduction from the initial dose (20). Therefore, we posit that treatment discontinuation, therapy modification, and tumor resection are integral components of managing the primary disease. Additionally, intentional dose omissions and unintentional dosing errors were predominantly associated with supply chain disruptions. Recognizing these distinctions is essential for holistic patient assessment and optimized therapeutic management.

Gamma-glutamyltransferase is one of the key enzymes involved in the transformation function of hepatocytes, and it is also an important link in glutathione metabolism (22). Therefore, we postulate that elevated gamma-glutamyltransferase levels may reflect pexidartinib-induced hepatotoxicity. Notably, decreased hemoglobin is a documented adverse reaction in the drug’s prescribing information. Significantly, we identified reduced serum iron and increased red cell distribution width as previously unreported AEs potentially causally linked to hemoglobin reduction. Increased blood calcium, an off-label AE of pexidartinib, may be associated with decreased phosphate mentioned in pexidartinib’s prescribing information (23, 24). The relationship between these clinical manifestations should attract the attention of patients and physicians, and the mechanism warrants further research.

Our analysis identified dysmenorrhea as a previously unreported adverse drug reaction associated with pexidartinib. This finding holds particular significance given the established association between anticoagulant-induced platelet dysfunction and menstrual abnormalities. A multicenter, single-arm, open-label, phase 2a, proof-of-concept trial (25) revealed that menorrhagia and dysmenorrhea are adverse drug reactions to rivaroxaban. Moreover, prolonged prothrombin time is detected as a kind of AE of pexidartinib. In a number of studies, prolonged prothrombin time is a common adverse reaction to tigecycline (26, 27). Therefore, the coagulation dysfunction induced by pexidartinib is worthy of attention and needs further exploration.

While leveraging that the FAERS database enables large-scale pharmacoepidemiologic surveillance, our study design inherits limitations inherent to spontaneous reporting systems that warrant cautious interpretation. First, because of the voluntary nature of the FAERS database, it has some inherent selection bias, such as the ethnicity and geography of the reported cases, the timing of approval and market penetration of different drugs, the level of public awareness of specific adverse reactions, and the fact that not all reports of serious adverse reactions occurring are being collected (28). Second, polypharmacy, comorbidities, and underlying disease severity pose a challenge in addressing confounding factors. The absence of comprehensive clinical information, such as interventions following AEs and the health status of the reporting patient, prevents the impact of confounding factors on the determination of causality between AEs and pexidartinib from being mitigated (29). Third, due to the absence of an accurate number of patients using pexidartinib, it remains impossible to calculate the true incidence rates for each AE (30).

Notwithstanding these limitations, the FAERS database remains a cornerstone resource in global pharmacovigilance, providing critical post-marketing surveillance insights through its unparalleled scale (30). It is crucial to acknowledge that although data mining techniques cannot compensate for the inherent limitations of a spontaneous reporting system, the combined utilization of the large-scale database and case reports remains an effective approach for delving into adverse drug reactions (31). The insights gleaned from the FAERS database provide valuable preliminary information for further investigation and prospective studies. Although the findings should be interpreted with caution, they represent contributions to a broader understanding of the safety profile of pexidartinib and its potential implications in clinical practice.

## 5 Conclusion

Through a comprehensive and systematic pharmacoepidemiologic study based on the FAERS database, we characterized the post-marketing safety profile linked to pexidartinib. Most of the AEs we identified closely align with the information provided in the FDA’s official prescribing guidelines. Notably, our study unveiled 16 unexpected AEs, expanding upon the existing knowledge derived from pre-marketing clinical trials. Although the limitations of the FAERS database are difficult to overcome, these findings are particularly valuable for ensuring drug safety. Further prospective clinical trials are warranted to establish a definitive connection between pexidartinib and these newly identified AEs. This study demonstrates the utility of pharmacovigilance databases in complementing clinical trial data for comprehensive drug safety assessment.

## Data Availability

The raw data supporting the conclusions of this article will be made available by the authors, without undue reservation.
