# Supplementary material for: Adverse events of pexidartinib for the treatment of TGCT: a real-world disproportionality analysis using FDA Adverse Event Reporting System database
Source: Front Oncol. 2025 Aug 18;15:1594585. doi: 10.3389/fonc.2025.1594585 (PMC12399690; doi:10.3389/fonc.2025.1594585)
Supplement: Supplementary file 1 [file Table1.docx]

| Table S1：Summary of major algorithms used for signal detection. | | | |
| --- | --- | --- | --- |
| **Algorithms** | **Indicator** | **Equation** | **Criteria** |
| ROR | ROR | ROR = ad/c/b | ROR05 > 1, N ≥ 2 |
|  |  | 95CI = e^ln(ROR)±1.96(1/a+1/b+1/c+1/d)^0.5^ |  |
| PRR | PRR | PRR = [a/(a+b)]/[c/(c + d)] | PRR≥2 |
|  | χ2 | χ2 = [(ad-bc)2 (a+b + c + d)]/[(a+b)(c + d)(a+c)(b + d)] | χ2 ≥ 4, N ≥ 3 |
| BCPNN | IC | IC = log2 [a (a+b + c + d)]/[(a+c)(a+b)] | IC025 > 0 |
|  |  | 95CI = e^ln(IC)±1.96(1/a+1/b+1/c+1/d)^0.5^ |  |
| MGPS | EBGM | EBGM = a (a+b + c + d)/(a+c)/(a+b) | EBGM05 > 2, N ≥ 0 |
|  |  | 95CI = e^ln(EBGM)±1.96(1/a+1/b+1/c+1/d)^0.5^ |  |

N, number of adverse event reports; CI, confidence interval; ROR, reporting odds ratio; ROR05, the lower limit of the 95 two-sided CI of the ROR; N, the number of co-occurrences; PRR, proportional reporting ratio; χ2, chi-squared; BCPNN, bayesian confidence propagation neural network; IC, information component; IC025, the lower limit of the 95 two-sided CI of the IC; MGPS, multi-item gamma Poisson shrinker; EBGM, empirical bayesian geometric mean; EBGM05, the lower 95 two-sided CI of EBGM.
